# TexSortML: A Dataset and Baseline Evaluation for Garment Classification in Automated Textile Sorting †

**DOI:** 10.3390/s26196236

**Published:** 2026-09-30

**Authors:** Julian Schanz, Martin Kohnle, Fabian Kopf, Sebastian Geldhäuser, Mesut Cetin, Alexandra Teynor

**Affiliations:** 1 Faculty of Computer Science, Technical University of Applied Sciences Augsburg, An der Hochschule 1, 86161 Augsburg, Germany; julian.schanz@tha.de (J.S.);; 2 Faculty of Mechanical and Process Engineering, Technical University of Applied Sciences Augsburg, An der Hochschule 1, 86161 Augsburg, Germany; martin.kohnle1@tha.de (M.K.); mesut.cetin@ita-augsburg.de (M.C.); 3 Institute for Textile Technology Augsburg, Am Technologiezentrum 5, 86159 Augsburg, Germany; sebastian.geldhaeuser@ita-augsburg.de

**Keywords:** automated textile sorting, machine learning, benchmark dataset, sensor-based sorting, conveyor belt, vision transformer, CLIP, circular economy

## Abstract

The growing volume of textile waste, driven by fast fashion and shortened garment lifespans, underscores the urgent need for scalable, automated sorting solutions. Progress is hindered by the absence of realistic, public datasets reflecting the variability of industrial textile sorting. We address this gap with TexSortML, a dataset of 751 post-consumer garments across 11 categories, captured under three controlled difficulty stages that simulate increasing handling disorder on a conveyor belt. On this dataset, we compare Convolutional Neural Networks (CNNs), Vision Transformers (ViTs), and CLIP-based zero-shot classifiers under five-fold cross-validation. A ViT fine-tuned directly on our data is the most accurate and most stable model at every stage, losing only 9.3 percentage points between the most and least controlled condition, against 16.6–16.9 points for the CNN baselines. The variant additionally pre-trained on external second-hand clothing data matches it only under controlled presentation and degrades sharply once garments are rotated or crumpled. CLIP-based zero-shot classifiers remain far behind and degrade the fastest. Rather than proposing a new architecture, this work contributes the dataset and a reproducible benchmark, and shows that extra training data can hurt when it is cleaner than the deployment condition. The dataset is publicly available on Zenodo.

## 1. Introduction

### 1.1. Problem Description

Europe produces an estimated 7–7.5 million tons of textile waste annually, yet only about 30–35% is collected, and just 1.4 million tons are currently sorted for reuse or recycling [1,2]. As most textiles are incinerated, landfilled, or exported [3,4], upcoming EU regulations, such as the Waste Framework Directive, will require higher collection rates, projected to reach 3.7 million tons by 2030, creating a sorting gap of around 2.3 million tons [1,5]. This underlines the urgent need for scalable, automated sorting solutions to handle growing waste streams driven by fast fashion. In automated sorting lines, garments are typically transported on conveyor belts.

The scope of this work is the garment as it presents itself on the conveyor belt, that is, its category, silhouette, and state of deformation, and not its material or fiber composition, which is the domain of spectroscopic sensing [6,7]. Both decisions are needed in an operational sorting line, but they are answered by different sensors; we isolate the visual component under the condition that makes it particularly challenging in practice, namely uncontrolled presentation. Material and fabric construction, such as woven versus knitted structures, also shape how a garment drapes and folds, but they are not annotated in this work.

Deep learning is well established in consumer-facing fashion applications such as category prediction, style classification, retrieval, outfit compatibility, quality control, and virtual try-on [8,9,10]. These systems operate on curated, high-resolution, front-facing imagery, supported by large-scale web-scraped datasets that reflect consistent lighting and clean backgrounds, and they achieve high accuracy under such idealized visual conditions [11]. Post-consumer garments on conveyor belts, however, appear crumpled, folded, or overlapping, making them harder to classify reliably [12,13], and distinguishing similar items, such as long-sleeve shirts versus lightweight jackets, remains challenging for current spectral or vision models under these uncontrolled conditions [12]. What matters for the task addressed here is the consequence: a garment has no clearly defined appearance on a conveyor belt.

In contrast to consumer fashion, the use of deep learning in textile recycling and reuse is still rare, fragmented, and underdeveloped [12,14]. Many efforts remain narrowly focused on quality control or fiber-level classification, often using near-infrared (NIR) spectroscopy [12,15]. While effective for detecting material composition, NIR-based systems are chemically selective but are inherently blind to the structural and visual cues required to judge garment integrity, wear condition, or reusability [6,7]. In particular, tasks such as distinguishing damaged items from resale-worthy ones, or interpreting garment type under deformation, lie entirely outside the scope of spectral methods [12]. Industrial vision systems have been proposed for sorting, but these are typically proprietary and rarely benchmarked under open, reproducible protocols [13]. Critically, almost no prior work has studied how fashion-trained or supervised vision models degrade under conveyor-induced distortions, or how zero-shot methods like CLIP behave in such settings.

### 1.2. Existing Datasets

The dataset landscape is accordingly skewed toward fashion and material classification [12]. Datasets such as DeepFashion [11], DeepFashion2 [16], and Fashion-MNIST [17] support style and attribute prediction on clean, centered images with full visibility and minimal ambiguity, and offer no relevance to physical sorting conditions; iMaterialist [18] and GLAMI-1M [19] extend these to multi-label and multilingual tasks but maintain the same static, consumer-aligned assumptions. Material-focused datasets like TextileNet [20] and FabricClassv2 [21] address textile fiber classification under lab conditions and are useful for composition analysis, but are not suitable for visual garment classification, reuse assessment, or operational sorting; NIR datasets similarly abstract away visual information [12]. Across both groups, none of these resources combines the properties that govern the behavior described above with images of whole garments: the fashion datasets carry no fiber labels, the material datasets label fibers but not garments in a handled state, and none of them records mass per unit area or any measure of fabric stiffness or elasticity. None of them therefore supports a prediction of how a given garment will present itself once it has been handled.

Recent efforts, such as the conveyor-belt dataset by Tian et al. (2024) [13], begin to reflect realistic capture conditions, including wrinkled and folded garments, as well as partial self-occlusions where one part of a garment obscures another. Nevertheless, these efforts remain rare, isolated, and non-standardized, and to our knowledge no openly accessible dataset currently captures garment classification under industrial conveyor conditions [12]. This work addresses that gap by introducing a benchmark dataset for garment category classification in textile sorting, collected under realistic conveyor belt conditions [22].

### 1.3. Challenges in Vision-Based Garment Classification

The model families that would be candidates for this task have themselves not been evaluated under these conditions. CNNs rely on spatially local filters and hierarchical features and are sensitive to the constrained feature visibility that folding, rotation, and self-occlusion produce [23,24,25]. Vision Transformers (ViTs) replace the local receptive field with global self-attention and carry fewer spatial biases [26]. Zero-shot vision-language models such as CLIP classify from natural language prompts without task-specific training [27,28,29]. Section 3 introduces each of the three in the form used here. What is missing is a rigorous comparison of them under real-world variability [12].

We frame the sorting task as a constrained domain generalization problem. We hypothesize that models trained on cleanly presented clothing imagery underperform on unstructured post-consumer garments without adaptation. To test this, we compare a controlled pair of Vision Transformers that differ only in an intermediate fine-tuning stage on cleanly presented clothing data, alongside CNN (ResNet) baselines and zero-shot CLIP prompting. The dataset captures garments in realistic states of deformation using structured acquisition protocols that create three distinct tiers of visual complexity, and the three model families are selected to span a spectrum of paradigms, from conventional supervised learning to prompt-based zero-shot inference, while aligning with methods either commonly applied, such as CNNs, or underexplored, such as ViTs and CLIP, in textile waste sorting.

### 1.4. Contribution

This article is an extended version of our contribution to the 11th Sensor-Based Sorting & Control Conference [30]. In summary, this paper contributes a realistic benchmark and a comparative evaluation of supervised and zero-shot approaches for robust garment category classification for textile sorting, supporting scalable, vision-based recycling systems for industrial deployment. Concretely, we release 2253 annotated frames of 751 post-consumer garments in 11 categories at three levels of handling disorder, evaluate CNN, ViT and zero-shot CLIP baselines under garment-level five-fold cross-validation, and show that intermediate fine-tuning on cleanly presented clothing data leaves the same architecture measurably worse under exactly the conditions a sorting line imposes.

## 2. Dataset

Due to the limited suitability of existing datasets for our application, this work focuses on developing and publicly releasing a custom dataset specifically tailored to garment classification under realistic conditions. The TexSortMLDataset can be obtained from Zenodo [22].

### 2.1. Data Collection

The dataset was developed in cooperation with a textile sorting facility in Germany, which provided garments and background information on its sorting operation. A total of 751 garments were sourced from post-consumer textile collection containers and made available by the facility. The garments were provided in boxes that had already been pre-sorted by the facility, so they are a sample of the material that passes through such an operation rather than a controlled draw from the incoming material flow. Whether their distribution matches that flow cannot be assessed, and the composition of collected textiles is also likely to vary over the year. The class frequencies reported in Table 1 therefore reflect what was available and are not balanced by design.

The eleven garment categories were defined by the authors, informed by that material and by the exchange with the facility. They were chosen to cover the most frequent item types among the available garments while remaining visually distinguishable, so that the label space is relevant to sorting and at the same time learnable from images. Each category is defined by observable visual and functional characteristics to ensure consistent and reproducible annotation. An overview of all defined categories, including item counts and criteria, is provided in Table 1. The category names follow sorting practice rather than consumer-facing usage; Table 1 therefore also lists, for each category, the most closely corresponding categories of DeepFashion [11] and DeepFashion2 [16].

### 2.2. Class Definition and Recycling Relevance

Background information from the cooperating facility indicates that distinguishing between upper-body garments (e.g., T-Shirts, Polo Shirts, Shirts, Longsleeves) matters in practice, because these types differ in reuse potential, market destination, and the way they are handled downstream. Polo Shirts and Shirts, for instance, are associated with higher-value resale streams, whereas generic T-Shirts are more often designated for downcycling or industrial reuse. Where possible, class boundaries therefore follow functional criteria such as collar type, closure, and outerwear function, which are the properties the sorting facility acts on.

This is also why the correspondence to public fashion taxonomies in Table 1 is deliberately loose. DeepFashion2, for example, separates upper garments primarily by sleeve length. Polo Shirt has no counterpart in either reference dataset and is absorbed into generic short- or long-sleeve tops, while Zipper/Sweat Jacket and Jacket/Coat both relate to Jacket but are separated here by outerwear function. Labels are therefore not interchangeable between the datasets without re-annotation. The schema is a pragmatic choice for this benchmark rather than a validated industrial taxonomy; Section 5.4 discusses what that implies for a deployed system.

### 2.3. Data Acquisition

Data were acquired at a dedicated recording station built to reproduce the geometry and the dynamics of an industrial sorting line under laboratory-grade repeatability. It comprises a motorized belt conveyor with adjustable speed, a high frame rate action camera mounted above the belt, and an enclosing light tent that decouples the capture volume from ambient illumination. Table 2 summarizes the components and their settings.

**Table 2:** Components and settings of the recording station. All recordings reported in this work were made with this configuration, so that imaging parameters and illumination are constant across garments, stages and recording sessions. The three belt speeds correspond to the difficulty stages defined in Table 3.

Component	Model/Specification	Setting
Camera	GoPro HERO11 Black, 1/1.9″ sensor (GoPro Inc., San Mateo, CA, USA)	1920 × 1080 (16:9), 240 fps, fixed focus, aperture and white balance, stabilization off
Conveyor drive	Laipple/KEB uniGear NMS 30 HU worm geared motor (KEB Antriebstechnik GmbH, Schneeberg, Germany)	Belt speeds 31/62/78 m/min (Stages 1 to 3)
Illumination	2 × LEANLED II, 2835 lm each, 5200 to 5700 K, CRI > 85 (LED2WORK GmbH, Pforzheim, Germany)	Approximately 5670 lm in total, continuous, diffuse via tent walls
Enclosure	Light tent, diffuse white interior	Blocks ambient light and specular reflections

**Table 3:** Definition of the three difficulty stages. The stages differ in belt speed and in the way the garment is placed on the belt, and together they span the range from a controlled, flat presentation to the handling disorder of an operational sorting line.

Difficulty Stage	Speed	Description
Stage 1	31 m/min	The garment is laid flat and neatly smoothed on the conveyor belt.
Stage 2	62 m/min	The garment is placed on the belt using one hand, resulting in minor rotation and displacement.
Stage 3	78 m/min	The garment is thrown onto the moving conveyor from a distance of one meter.

The belt is driven by a worm-geared motor with the speed set manually on the drive controller. Three belt speeds of 31, 62 and 78 m/min were used and are tied to the difficulty stages described in Section 2.4. The belt surface is dark green and matte, which separates it from the majority of garment colors in the material flow and keeps the background constant across all recordings. Garments close to the belt color were rare in the recorded material (4 of 751 garments); since none of the evaluated models relies on background segmentation, low garment–belt contrast reduces the visual separation but does not prevent classification.

Recording used a GoPro HERO11 Black at 1920 × 1080 pixels (16:9) and 240 frames per second, the highest frame rate the camera provides at this resolution, with the Linear digital lens, which removes the barrel distortion of the wide setting and preserves straight edges across the belt. The camera was mounted rigidly above the belt at a fixed working distance of 1.2 m and oriented perpendicular to the belt surface, so that its field of view covers the full belt width. Focus, aperture, white balance and exposure were fixed for all recordings, and electronic stabilization was disabled, since the camera is static and stabilization would introduce a frame-dependent geometric transformation. The frame rate follows from the belt speed rather than from a slow-motion intent: at 240 fps the inter-frame interval is 4.17 ms, corresponding to a garment displacement of 2.2 mm at 31 m/min, 4.3 mm at 62 m/min and 5.4 mm at 78 m/min. Individual frames therefore remain sharp even at the highest stage, and every garment yields enough candidate frames for the selection described in Section 2.5.

The capture area is enclosed by a light tent with diffuse white inner walls and illuminated by two LED machine luminaires mounted centrally above it, so that light reaches the garment largely indirectly via the tent walls (Table 2). The light tent was introduced in response to a problem observed during initial trials. Without it, the ambient hall lighting reflected off garment surfaces, in particular smooth and coated fabrics, and off the belt itself, producing specular highlights that locally saturated the sensor and erased texture and contour information in the affected regions. Because these highlights move with the garment and depend on its instantaneous orientation, they affect the difficult stages most, that is, precisely those the dataset is built to capture. Enclosing the capture volume removes the direct reflection paths and replaces variable hall illumination with a constant, diffuse light field. All recordings reported here were made inside the tent, so illumination is constant across garments, stages and recording sessions.

### 2.4. Difficulty Stages

In conjunction with the adjustable speed settings of the conveyor belt, three distinct scenarios, referred to as stages, were defined to capture the behavior of garments under varying dynamic conditions. Each garment was recorded across all three stages to introduce meaningful variability into the dataset. Stages are differentiated according to the speed of the conveyor and the way the garment is placed on the belt as described in Table 3.

**Figure 1:**
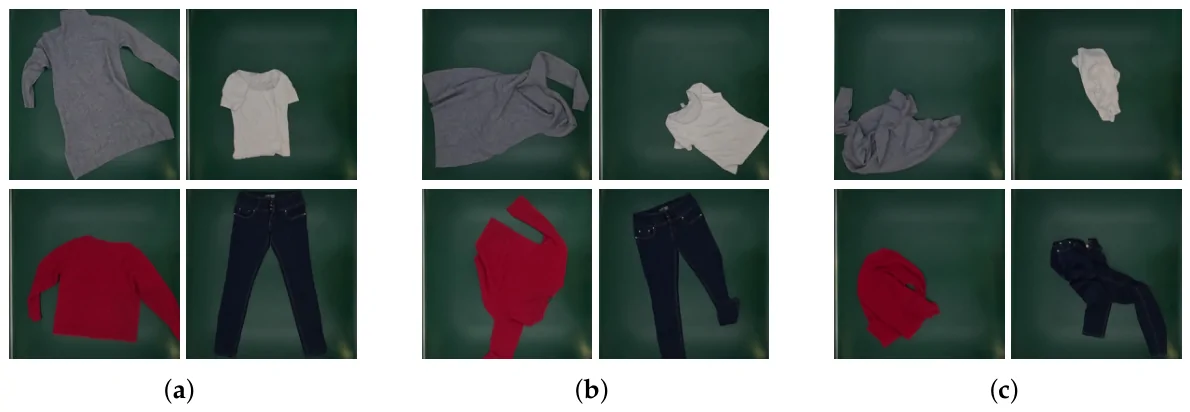
Exemplary representations of four different garments across all three defined stages. (**a**) Stage 1: Garment laid flat and aligned, (**b**) Stage 2: Garment placed with one hand, showing rotation, (**c**) Stage 3: Garment thrown from a distance of 1 m.

In Figure 1a–c, four example garments from each of the three defined stages are illustrated. These examples highlight the variation in garment position, folding, and deformation that arises from different handling and conveyor belt dynamics.

Across all three stages, garments are recorded individually, with exactly one item present in the camera’s field of view at any time. The dataset therefore captures self-occlusion caused by folding, crumpling, and rotation of a single garment, but does not include inter-object occlusion from multiple overlapping garments, even under the most challenging Stage 3 conditions.

### 2.5. Frame Extraction and Dataset Splitting

After data acquisition, individual image frames were extracted from the recorded videos and labeled according to their textile category and difficulty level. Each video stream corresponded to a specific category, simplifying the labeling process. Garments were recorded in the same order across all three stages to allow for consistent matching.

For each garment, a single frame was selected manually from the recorded video, at the moment the garment was fully visible within the camera’s field of view and no part of it was cut off by the image border. The selection was made by one annotator following this criterion; no automatic frame scoring was applied. This yields 2253 annotated frames (751 garments × 3 stages) that serve as the input to all experiments. The frames are released at the full recording resolution of 1920 × 1080 pixels.

Since only 751 garments are available, we avoid relying on a single fixed split and instead evaluate all experiments with stratified five-fold cross-validation: the 751 garments are partitioned into five folds, each supervised model is trained on four folds and evaluated on the held-out fold, and every reported value is the mean over the five held-out folds with its standard deviation. The zero-shot CLIP variants are not trained and are scored on the same five held-out folds, so that all reported results refer to identical evaluation sets (Section 3.4). Folds are formed at the level of garments rather than frames: all images of a given garment, across all three difficulty stages, are assigned to the same fold, so that no garment appears in both the training and the evaluation partition of a run. Folds are stratified by garment category to preserve the class distribution of Table 1.

## 3. Methods

**Figure 2:**
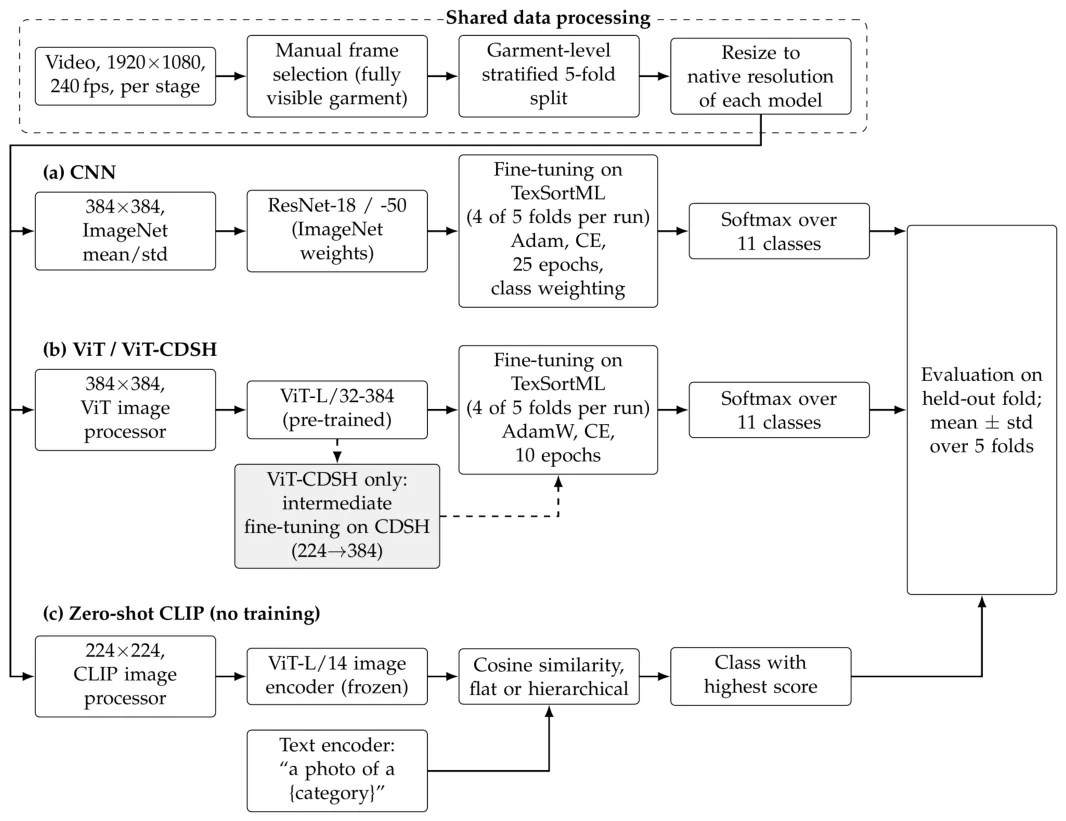
Processing chain of the three model families. All frames pass through the same data processing before they are resized to the native input resolution of each model. The supervised models (**a**,**b**) are fine-tuned on four folds, ViT-CDSH after an intermediate fine-tuning stage on CDSH; the zero-shot models (**c**) are not trained. All models are evaluated on the same held-out folds.

We compare three approaches to garment classification: supervised learning with Convolutional Neural Networks, supervised learning with Vision Transformers, and zero-shot classification using CLIP. Figure 2 shows the processing chain of all three. All models receive the frames described in Section 2.5, resized by their respective image processor to the input resolution given below.

### 3.1. Convolutional Neural Networks (CNNs)

In the supervised learning framework, ResNet architectures were evaluated to establish a performance baseline using labeled garment images. Two variants, ResNet-18 (referred to as RN-18) and ResNet-50 (referred to as RN-50), initialized with ImageNet-pretrained weights, were fine-tuned on our labeled dataset at an input resolution of 384 × 384 pixels, identical to that of the Vision Transformers.

Preprocessing involved input normalization following the standard ImageNet protocol, using channel-wise mean and standard deviation values tailored to the ResNet architectures.

Model training was implemented in PyTorch (version 2.9.0, CUDA 12.8) [31], employing a cross-entropy loss function and the Adam optimizer with a learning rate of $10^{-4}$, a batch size of 32 and 25 epochs. Learning rate and number of epochs were chosen empirically in preliminary runs on the same dataset and then held fixed across all folds and stages; no per-fold hyperparameter selection was performed. No regularization such as weight decay or dropout was added beyond the default settings of the respective optimizer and pretrained model. Class imbalance was addressed through class weighting in the loss function.

### 3.2. Vision Transformers (ViT)

Alongside CNNs, supervised learning was applied to two Vision Transformer (ViT) models [26], each with an input resolution of 384 × 384 pixels. In one experiment, we used google/vit-large-patch32-384 [32] as the base model and fine-tuned it directly on our labeled dataset (referred to as ViT in the remainder of this paper). In a separate experiment, we first fine-tuned the base model on the Clothing Dataset Second-Hand (CDSH) [33], a large curated dataset of second-hand garments collected at commercial sorting facilities, using the processed release of [34] (43,132 images, backgrounds removed, resized to 224 × 224 pixels, with every garment presented flat, fully visible and canonically oriented), and then fine-tuned it further on our labeled dataset (referred to as ViT-CDSH in the remainder of this paper). Both Vision Transformer variants are fine-tuned on our labeled dataset and differ only in whether the intermediate fine-tuning stage on CDSH preceded this final fine-tuning. For the intermediate fine-tuning stage, the 224 × 224 images were upsampled to the model’s native input resolution of 384 × 384, so that both fine-tuning stages operated at identical input dimensions.

The preprocessing and normalization were adapted to match the architectural specifications, using the ViTImageProcessor [35] to ensure consistency with the respective pre-training configurations.

Training settings for the ViT models used the AdamW optimizer with cross-entropy loss, a learning rate of $10^{-4}$, a batch size of 32 and 10 epochs. These values were determined in the same way and likewise held fixed across all folds.

### 3.3. Zero-Shot Approach

CLIP enables image classification through natural language prompts without task-specific fine-tuning and supports both Vision Transformer and ResNet-based image encoders.

The model encodes both input images and textual prompts into a shared embedding space. Class predictions are computed by measuring cosine similarity between the image embedding and a set of candidate class prompts. Each category is represented by one prompt formed from its category name in the fixed template “a photo of a {category}”. No alternative formulations were evaluated. The hierarchical strategy described below additionally uses prompts for the intermediate groupings. Two prompting strategies were implemented:

- **Flat Prompting Strategy (referred to as CLIP-Plain):**
  All class prompts are evaluated simultaneously, and the class with the highest similarity score is selected. This approach enables fast batch inference but assumes sufficient separation between all label embeddings.
- **Decision Tree Prompting Strategy (referred to as CLIP-Dec):**
  A hierarchical scheme organizes prompts into semantic levels (e.g., upper vs. lower garments, followed by more specific comparisons such as “shirt” vs. “jacket”). Temporary groupings, such as “collared tops” or “sleeveless garments”, help increase decision margins before final class assignment.

### 3.4. Evaluation Protocol

All experiments follow the same five-fold cross-validation scheme described in Section 2.5, so that supervised and zero-shot results are directly comparable. Each difficulty stage is treated as a separate experiment: models are trained and evaluated on frames of the same stage only, so that each run uses approximately 600 training and 150 evaluation frames. For the supervised models (RN-18, RN-50, ViT, ViT-CDSH), each fold yields an independently initialized training run on four folds and an evaluation on the held-out fold; the training parameters given in Section 3.1 and Section 3.2 are held fixed across folds and no per-fold hyperparameter selection is performed.

The CLIP variants involve no parameter updates, so their fold-to-fold variation does not originate from training. Applying the identical partition to them nonetheless serves two purposes: the zero-shot models are scored on exactly the same held-out garments as the supervised ones, and the resulting standard deviation quantifies how much the reported accuracy depends on which garments happen to be evaluated. This separates variance caused by dataset composition from variance caused by training, and it is the reason the CLIP standard deviations in Table 4 are of the same order as the supervised ones despite the absence of learning.

Accuracy is reported as the mean over the five held-out folds with the standard deviation across folds. Comparisons between models are made on the same folds.

## 4. Results

Table 4 summarizes all experiments. Two observations structure the discussion that follows: the ranking of model families changes with the degree of handling disorder, and the two ViT variants, identical in architecture and differing only in whether they were fine-tuned on an external clothing dataset beforehand, separate more strongly than any two different architectures.

### 4.1. Stage 1

Under controlled presentation, all supervised models perform well and the differences between them are small relative to the fold-to-fold spread. ViT reaches the highest mean accuracy (95.23%), but its lead over ViT-CDSH (94.58%) and RN-50 (93.00%) is within one standard deviation and should not be read as a ranking. The meaningful gap at this stage is between the supervised models and the zero-shot baselines, which trail the supervised models by 12 to 17 percentage points depending on the pairing, and the best supervised model by roughly 17 points.

### 4.2. Stage 2

Introducing rotation and displacement separates the models. ViT loses 3.3 points and stays highest at 91.90%, RN-50 loses 4.9 points, and RN-18 loses 8.9 points. ViT-CDSH, however, drops by 25.1 points to 69.52%, below both CNN baselines and 22.4 points below the architecturally identical ViT. Since the two ViT variants share architecture, input resolution and training schedule, and differ only in the intermediate fine-tuning stage, this gap is attributable to that stage rather than to the backbone.

### 4.3. Stage 3

Under the most severe deformation, the ordering of Stage 2 is preserved, and the distances between ViT and all other models except ViT-CDSH widen. ViT retains 85.94% and shows the flattest overall degradation of all models (9.3 points from Stage 1), while RN-50 and RN-18 lose 16.6 and 16.9 points, respectively. ViT-CDSH remains at the level it fell to in Stage 2 (68.93%). Both CLIP variants approach the range where a substantial share of predictions is no longer informative, losing between 31.0 and 36.4 points relative to Stage 1.

### 4.4. Prompting Strategies

Across the three stages, flat prompting is ahead of hierarchical prompting by 0.6 to 6.0 percentage points; only at Stage 3 does the difference exceed the fold-to-fold spread. We therefore find no advantage of hierarchical over flat prompting on this dataset.

### 4.5. Class-Level Behavior

**Figure 3:**
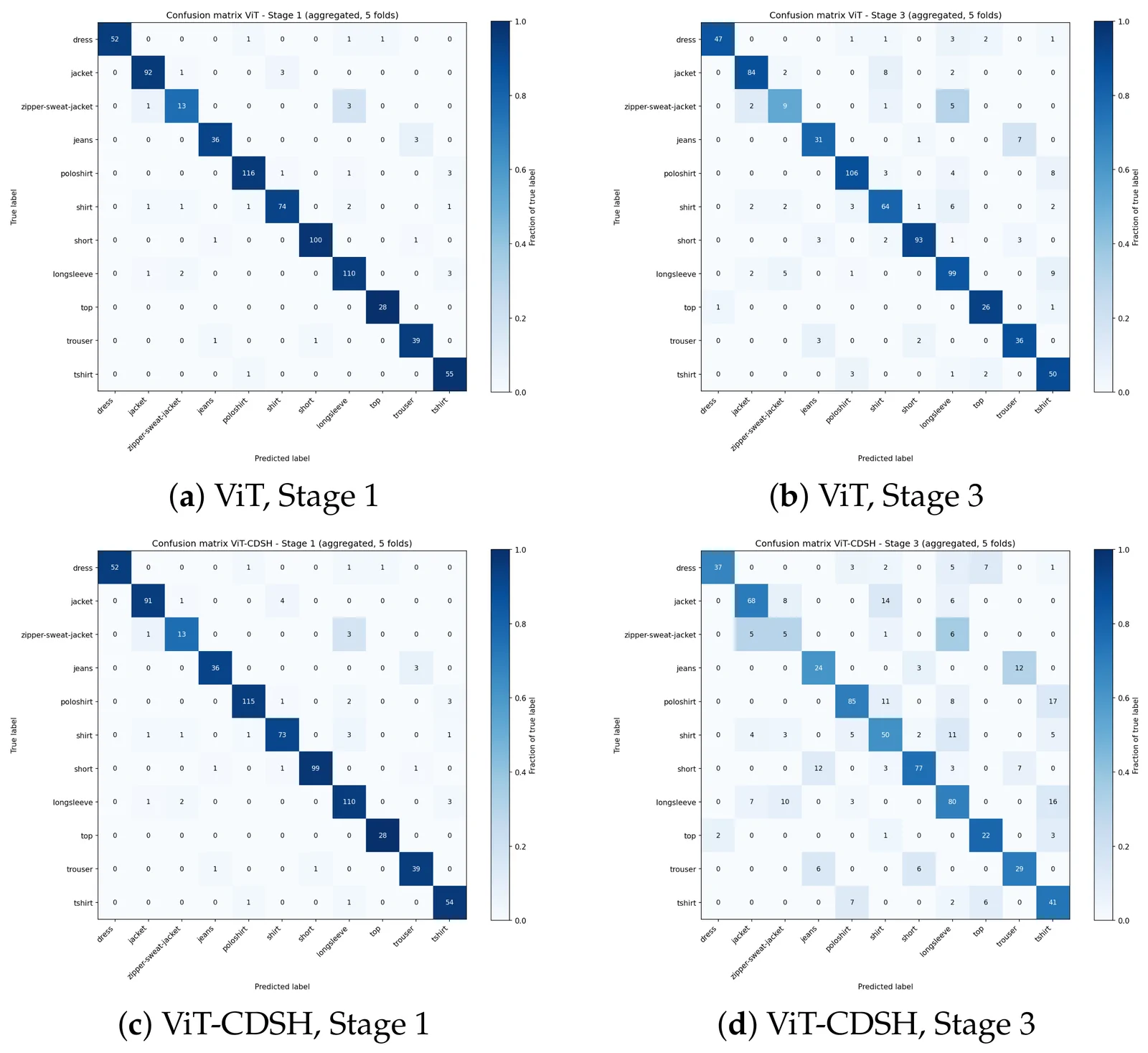
Confusion matrices for the two Vision Transformer variants under the most and the least controlled presentation, aggregated over the five held-out folds so that every garment is counted exactly once. Cell values are counts; the color encodes each cell as a share of its row total, and row totals are the item counts of Table 1. (**a**,**b**) show the ViT baseline, (**c**,**d**) the variant with the intermediate fine-tuning stage; the corresponding accuracies are given in Table 4.

To show where the accuracy is lost, Figure 3 gives the confusion matrices of the two Vision Transformer variants, aggregated over the five held-out folds so that every garment is counted exactly once. Each row corresponds to the true category and each column to the predicted one, so correct classifications lie on the diagonal and every off-diagonal cell counts garments of one category assigned to another. We restrict the figure to ViT and ViT-CDSH because they differ only in the intermediate fine-tuning stage, so any difference between their matrices is attributable to that stage, and to Stage 1 and Stage 3 because these mark the most and least controlled presentation.

The degradation is broad rather than concentrated: at Stage 3, ViT-CDSH classifies fewer garments correctly than ViT in every one of the eleven categories, and for neither model is the loss carried by a few categories, so neither fails on a particular kind of garment. The exception is Zipper/Sweat Jacket, the smallest category with 17 items, which is already the weakest under controlled presentation (13 of 17 for both variants) and falls to 9 and 5, respectively, at Stage 3, confused almost exclusively with Jacket/Coat and Longsleeve. Category size alone does not account for this, as Top is comparably small with 28 items and is still recognized 26 and 22 times under Stage 3. The confusions that do occur remain within the coarse structure of the label space: of the 106 and 233 misclassified garments at Stage 3, only 4 and 8, respectively, cross the boundary between upper-body and lower-body garments.

Because overall accuracy is dominated by the larger categories, Table A1 in Appendix A additionally reports macro- and support-weighted precision, recall and F1 for all models, derived from the confusion matrices aggregated over the five held-out folds. The macro average weights every category equally. Macro-F1 lies 1.3 to 6.5 points below accuracy, but the ranking of the models at every stage is the same as in Table 4. For the two ViT variants, the difference is 1.3 to 3.8 points, and at Stage 3 the gap between them widens slightly from 16.9 points in accuracy to 17.9 points in macro-F1. Zipper/Sweat Jacket is the weakest category by F1 in every condition (76.5 for both ViT variants at Stage 1; 51.4 and 23.3 at Stage 3).

## 5. Discussion

Two results deserve interpretation beyond the ranking they produce: the comparatively flat degradation of the ViT baseline, and the failure of its externally fine-tuned counterpart under exactly the conditions the dataset was built to capture.

### 5.1. Graceful Degradation of Attention-Based Models

Attention-based models degrade more gracefully than the CNN baselines. Across the three stages, ViT loses 9.3 percentage points while RN-50 and RN-18 lose 16.6 and 16.9. A plausible reading is that the convolutional baselines rely on hierarchies of spatially local features whose composition assumes an approximately canonical layout, whereas global self-attention can relate distant garment parts, such as a sleeve and a collar that folding has brought close together, without depending on their expected relative position. We note that this is an interpretation of an observed accuracy pattern, not a mechanistic claim; attribution studies on this dataset would be required to test it.

The comparison is also not controlled for model size. All supervised models receive 384 × 384 inputs, but with 305.6 million parameters and about 45.3 GMACs per image, the ViT is roughly 13 times larger than RN-50 (23.5 million, 12.1 GMACs) and 27 times larger than RN-18 (11.2 million, 5.3 GMACs). Part of its advantage may therefore stem from capacity rather than from self-attention itself. The difference also matters for deployment: on embedded hardware at the sorting line, where memory, power and latency are limited, a ResNet is considerably easier to run. Whether a ResNet becomes sufficient once training data are recorded under the handling conditions of the target line is left to future work (Section 6). Inference latency on specific embedded platforms was not measured and remains part of a deployment study.

### 5.2. Negative Transfer from Cleanly Presented Source Domains

Intermediate fine-tuning on a cleanly presented source domain is harmful under handling disorder. ViT and ViT-CDSH share architecture, resolution, optimizer and schedule; they differ only in one preceding fine-tuning stage on a large second-hand clothing dataset. Under controlled presentation, the two are indistinguishable, but as soon as garments are rotated or crumpled the externally fine-tuned variant falls 22.4 and 17.0 points behind, and below both CNN baselines. The source dataset shows garments flat, fully visible, canonically oriented and with backgrounds removed, and, being upsampled from 224 × 224, it also differs from our frames in effective resolution. Fine-tuning on it therefore rewards exactly the cues that Stage 2 and Stage 3 destroy (overall silhouette, complete outline, expected part layout), and appears to do so at the cost of the broader invariances the ImageNet-pretrained backbone provides.

The class-level errors support this reading. At Stage 3, the additional misclassifications of ViT-CDSH concentrate on pairs that share a silhouette and differ mainly in local cues: Polo Shirt predicted as T-Shirt (17 vs. 8 for ViT), Jacket/Coat as Shirt (14 vs. 8), Jeans as Trousers (12 vs. 7) and Shorts as Jeans (12 vs. 3), that is, distinctions that rest on collar, closure, fabric texture or length (Figure 3). A model that has learned to rely on the complete, canonically oriented outline loses exactly these distinctions once that outline is no longer available. This explanation is inferred from accuracy and error patterns; attention or attribution maps were not computed and would be required to confirm it.

This reading predicts that the gap between the two variants should narrow once disordered presentations are part of the target training data. Testing it requires training on all three stages jointly while holding the training set size fixed, since otherwise distribution and scale change at the same time. We outline this experiment in Section 6.

### 5.3. Zero-Shot CLIP Without Domain Adaptation

Zero-shot CLIP remains unsuitable without adaptation. Both prompting strategies trail the supervised models by wide margins at every stage and degrade the fastest of all methods (31.0 and 36.4 points). Two explanations are possible. The first concerns how the classes are named rather than where their boundaries lie (Section 2.2): the eleven categories are named after industrial sorting practice rather than after the consumer vocabulary that dominates CLIP’s web-scale training data. The second concerns the images: the visual representation may not be robust to the deformations of Stages 2 and 3. The per-class results locate the failure more precisely than either attribution does. Under the most severe handling disorder, the zero-shot predictions concentrate on a single category: at Stage 3, Longsleeve receives 2.4 predictions per actual item under flat prompting and absorbs the majority of all T-Shirts and three quarters of all Zipper/Sweat Jackets, while the smallest categories receive almost no predictions at all. Aggregate accuracy conceals this, because the high recall of the attracting category and the low recall of every other category offset each other. Under hierarchical prompting, the predictions accumulate on Shirt instead, at 2.8 per actual item, and that strategy yields no consistent improvement over flat prompting either. Prompt structure, at least in the two forms tested here, does not compensate for the absence of domain adaptation.

### 5.4. Industrial Relevance of the Category Schema

A benchmark is only as useful as the label space it commits to, and the schema used here has clear limitations. Industrial sorting facilities operate with up to several hundred categories [36], so eleven classes are a coarse subset, and a deployed system would need to refine within them or use a larger label space. The schema covers category only and records neither condition, damage, brand, or size, which co-determine the destination of an item in reuse grading, nor fabric construction (Section 5.5). The garments were provided pre-sorted (Section 2.1), so the class frequencies in Table 1 do not necessarily represent the composition of an operational material flow. Finally, all garments originate from one facility in one national market, so transfer of the label space to other facilities remains to be shown.

### 5.5. Scope: Garment Presentation Versus Material Composition

TexSortML labels what a garment is and how it is presented, not what it is made of or in which condition it is. This boundary is not strict: woven and knitted fabrics, or heavy and light materials, drape, fold and crumple differently on the belt. Because these properties are not annotated, part of the variation within a class under Stages 2 and 3 may stem from them rather than from the garment category. In an operational line, composition is determined by NIR spectroscopy [6,7], which is blind to garment category and deformation, while the visual models studied here are blind to composition. Fusing both measurements of the same item, and annotating fiber type and fabric construction in future recordings, would let one sorting decision draw on both and make the influence of material on presentation measurable.

### 5.6. Limitations

The dataset comprises 751 garments recorded at three difficulty stages, which is small compared with general-purpose vision benchmarks and reflects the cost of physically recording individual post-consumer items on a conveyor system. Five-fold cross-validation mitigates the consequences of that size for evaluation, since every garment is used for evaluation exactly once and the reported standard deviations make the remaining uncertainty explicit, but it does not increase the diversity of the underlying material. The class distribution is unbalanced (Table 1), ranging from 17 to 121 items per category, so aggregate accuracy is dominated by the larger ones; the macro-averaged results in Table A1 weight every category equally. The confusion matrices of the two ViT variants (Figure 3) show that size is not the only factor at work: Top, with 28 items, reaches the highest recall of all categories at both Stage 1 and Stage 3, whereas Zipper/Sweat Jacket, with 17, is the weakest by a wide margin, plausibly because few examples coincide with a boundary that rests on closure type rather than on overall shape. Either way, the estimates for the smallest categories rest on few garments and should be read accordingly.

The recordings contain exactly one garment in the field of view at a time and were captured in a light tent, whose constant, diffuse illumination was necessary to suppress the specular highlights observed without it, but which is more controlled than the lighting of a typical sorting hall. Inter-object occlusion from overlapping garments and variable lighting, both common in production sorting lines, are consequently outside the scope of this benchmark. The frame used for each garment was selected manually rather than by an automatic criterion, so the choice of frame within a recording reflects one annotator’s judgment; the raw video streams are part of the released dataset, so alternative selection strategies can be derived from the same material. Finally, all recordings originate from a single facility and a single camera setup; generalization across facilities remains untested.

No systematic hyperparameter search or sensitivity analysis was performed; each model family uses one fixed configuration across folds and stages, and the preliminary runs used to fix the learning rate and the number of epochs were carried out on the same data. Other settings may shift individual accuracies. The gap between the two ViT variants is nevertheless unlikely to be an artifact of the shared configuration: under identical settings both reach the same accuracy at Stage 1 (95.2% and 94.6%), and they diverge only once handling disorder is introduced.

## 6. Conclusions and Future Work

This study evaluated baseline deep learning architectures with respect to their suitability for automated garment classification. By employing scenarios with increasing image complexity, a nuanced assessment of each model’s generalization capabilities was made possible.

Overall, the ViT fine-tuned directly on our data proved the most accurate and most stable model across all levels of difficulty, degrading by 9.3 percentage points between the most and least controlled condition, compared with 16.6 and 16.9 points for the CNN baselines. The variant additionally fine-tuned on a large external second-hand clothing dataset matched it only under controlled presentation and fell behind both CNN baselines once garments were rotated or crumpled, showing that additional in-domain data can be harmful when its presentation is cleaner than the deployment condition. The zero-shot CLIP classifiers remained far behind the supervised models at every stage and degraded the fastest, so they are not suitable for this task without domain adaptation.

Several directions for future research emerge from these findings:

- **Data augmentation and expansion**: A larger and more diverse dataset would broaden the range of garment types, fabrics and deformation patterns represented, which the cross-validation protocol adopted here makes measurable but cannot substitute for. Targeted augmentation such as rotation, self-occlusion or perspective distortion could further improve robustness.
- **Presentation-aware domain adaptation**: Our results indicate that additional in-domain data do not necessarily help, and can hurt, when their presentation is cleaner than the deployment condition. A promising direction is therefore not more clothing data, but source data or augmentation that reproduces the deformation statistics of the conveyor, such as synthetic folding and crumpling, physically based cloth simulation, or augmentation policies fitted to the Stage 2 and Stage 3 distributions.
- **Further experiments**: All models in this study were trained and evaluated within a single difficulty stage. Training on combinations of stages, for instance only on the disordered Stages 2 and 3, would show the amount of disordered training data needed and whether the gap between ViT and ViT-CDSH narrows once such presentations are part of the training data (Section 5.2). In such experiments, the training set size must be held fixed, since distribution and scale otherwise change together. Ablations, for example, of the intermediate fine-tuning stage or of model size, would further separate the factors behind the observed differences and show whether a ResNet is sufficient for deployment at a fraction of the computational cost.
- **Combining model types through ensembling**: Ensemble strategies could integrate the strengths of different architectures, for instance by combining the low computational cost of CNNs with the robustness of fine-tuned Transformers.
- **Extended use of the recorded dataset**: The publicly released dataset, consisting of both extracted images and the original video streams, presents opportunities beyond static image classification. Future work could explore video-based approaches that leverage temporal information, such as garment motion patterns or transitions between frames, to improve classification accuracy or enable new tasks like garment tracking, segmentation, or dynamic context analysis.

The results emphasize the importance of task-specific model adaptation and the careful selection of data-related strategies in developing effective visual classification systems. The introduced dataset plays a key role in enabling reproducible research and opens new avenues for the exploration of temporal and multimodal learning in the field of automated garment analysis.

## Figures and Tables

**Table 1:** Overview of garment categories, item counts, category descriptions, and corresponding category names in the DeepFashion [11] and DeepFashion2 [16] datasets. The listed names indicate corresponding or closely related categories in the two reference datasets and are provided for orientation. The annotation scheme solely used the names in the first column.

Category	Count	Description	DeepFashion/DeepFashion2
T-Shirt	56	Short-sleeved upper garment, sleeves shorter than elbows, no collar	Tee, short-sleeve top
Polo Shirt	121	With polo collar, available with short or long sleeves	–
Shirt	80	Collared garment with buttons, includes short and long sleeves	Shirt, Button-Down, Flannel
Longsleeve	116	Long-sleeved upper garment, without collar, closed front, sleeves longer than elbows	long sleeve top
Jacket/Coat	96	Fully openable at the front, equipped with buttons or zippers, typically worn as outerwear	Jacket, Coat, short-sleeve outerwear, long-sleeve outerwear
Jeans	39	Long trousers made of denim fabric, often with rivets	Jeans
Trousers	41	Long pants made of any fabric other than denim	Pants, trousers
Shorts	102	Short trousers, any fabric, typically knee-length or shorter	Shorts
Dress	55	One-piece long garment, typically covering torso and legs	Dress, short sleeve dress, long sleeve dress, vest dress, sling dress
Zipper/Sweat Jacket	17	Partially or fully openable upper garment, includes zippers or no closure, not classified as outerwear, may include hood, long sleeves	Hoodie, Jacket
Top	28	Sleeveless or short-sleeved women’s upper garment	Top, Tank, short sleeve top, vest, sling

**Table 4:** Model performance across the three difficulty stages. Values are mean accuracy over five folds in percent, with the standard deviation across folds. The last column reports the absolute change from Stage 1 to Stage 3 in percentage points. Bold marks the highest mean per stage and the smallest loss; differences within one standard deviation should not be read as a ranking.

Model	Stage 1	Stage 2	Stage 3	Δ S1→S3
RN-18	89.91 ± 3.68	81.05 ± 3.29	73.02 ± 2.27	−16.9
RN-50	93.00 ± 2.27	88.13 ± 3.06	76.44 ± 2.94	−16.6
ViT	**95.23 ± 1.63**	**91.90 ± 1.96**	**85.94 ± 1.17**	**−9.3**
ViT-CDSH	94.58 ± 2.34	69.52 ± 1.91	68.93 ± 1.12	−25.7
CLIP-Plain	78.32 ± 2.11	61.45 ± 4.45	47.34 ± 4.45	−31.0
CLIP-Dec	77.75 ± 2.97	58.87 ± 1.63	41.34 ± 5.07	−36.4

## Data Availability

The TexSortML dataset, including the extracted frames and the raw video recordings, is openly available on Zenodo at https://doi.org/10.5281/zenodo.22143896.

## Abbreviations

CLIP	Contrastive Language–Image Pretraining
CNN	Convolutional Neural Network
ML	Machine Learning
NIR	Near-Infrared
RN-18	ResNet-18
RN-50	ResNet-50
ViT	Vision Transformer
ViT-CDSH	Vision Transformer–Clothing Data Second-Hand

## Appendix A. Macro-Averaged and Support-Weighted Results

Table A1 complements the accuracies in Table 4 with macro- and support-weighted precision, recall and F1 for all models and stages.

**Table A1:** Macro-averaged and support-weighted precision (P), recall (R) and F1 (%) for all models, computed from the confusion matrices aggregated over the five held-out folds. The support-weighted recall equals the overall accuracy on all 751 garments; it can differ from the fold-averaged accuracy in Table 4 by up to 0.07 points because the folds are not exactly equal in size (751 garments, stratified by category).

		Macro			Support-Weighted		
Model	Stage	P	R	F1	P	R	F1
RN-18	1	87.44	87.41	87.29	90.21	89.88	89.94
RN-18	2	76.34	77.21	76.40	82.02	81.09	81.26
RN-18	3	68.89	69.18	68.53	75.06	72.97	73.58
RN-50	1	91.02	91.16	91.02	93.08	92.94	92.96
RN-50	2	85.22	85.35	85.12	88.48	88.15	88.18
RN-50	3	72.12	72.82	72.04	78.08	76.43	76.89
ViT	1	93.58	93.98	93.74	95.30	95.21	95.22
ViT	2	89.53	89.71	89.50	92.05	91.88	91.88
ViT	3	82.92	83.59	83.04	86.61	85.89	86.07
ViT-CDSH	1	93.12	93.44	93.24	94.65	94.54	94.56
ViT-CDSH	2	64.24	65.18	63.87	72.10	69.51	70.18
ViT-CDSH	3	65.63	66.25	65.18	71.91	68.97	69.83
CLIP-Plain	1	74.05	74.53	73.91	79.63	78.30	78.63
CLIP-Plain	2	55.94	55.65	54.87	64.51	61.38	62.22
CLIP-Plain	3	47.26	45.60	45.20	53.05	47.40	49.18
CLIP-Dec	1	73.55	74.11	73.46	79.13	77.76	78.13
CLIP-Dec	2	53.56	53.29	52.46	62.14	58.85	59.74
CLIP-Dec	3	42.19	40.09	39.71	47.36	41.28	43.13
